# Substance Use and Mental Health Outcomes for Comorbid Patients in Psychiatric Day Treatment

**DOI:** 10.4137/sart.s3462

**Published:** 2009-10-28

**Authors:** Stephen Magura, Andrew Rosenblum, Thomas Betzler

**Affiliations:** 1 The Evaluation Center, Western Michigan University, Kalamazoo, MI.; 2 National Development and Research Institutes, New York, NY.; 3 Albert Einstein College of Medicine, New York, NY. Email: stephen.magura@wmich.edu

**Keywords:** substance abuse, psychiatric treatment, treatment outcomes, comorbidity, co-occurring disorders

## Abstract

The study’s purpose was to determine treatment outcomes for patients who present with drug use vs. those presenting with no drug use at admission to a psychiatric day treatment program. Consecutively admitted patients completed confidential interviews which included psychological distress and quality of life measures and provided urine specimens for toxicology at admission and six month follow-up. Subjects positive by past 30 day self-report or urinalysis were categorized as drug users. Major psychiatric diagnoses were: major depression 25%; bipolar, 13%; other mood 13%; schizoaffective 13%; schizophrenia 13%. Drug use at admission was: cocaine 35%; marijuana 33%; opiates 18%, (meth)amphetamines, 6% For each of these drugs, the percentage of patients positive at admission who remitted from using the drug significantly exceeded the percentage negative at baseline who initiated using the drug. Overall, there were significant decreases in psychological distress and significant improvement on quality of life, but no change on positive affect. There were no significant differences between drug users and non-drug users on symptom reduction and improvement in quality of life. Psychiatric day treatment appears to benefit comorbid patients by reducing the net number of patients who actively use certain common drugs and by improving psychological status and quality of life to the same degree as for non-drug using patients.

## Introduction

In 2007, according to the National Survey of Drug Use and Health (NSDUH), there were an estimated 24.3 million adults aged 18 or older in the United States with Serious Psychological Distress (SPD) in the past year. This represents 10.9 percent of all adults in the USA. Meeting the criteria for SPD indicates that the respondent endorsed having symptoms at a level known to be indicative of having a mental disorder (i.e. any disorder such as an anxiety or mood disorder). SPD in the past year was associated with past year substance dependence or abuse. Among adults with SPD, 22.1 percent were dependent on or abused illicit drugs or alcohol. The rate of illicit drug dependence or abuse among adults without SPD was 7.6 percent.^1^ The U.S. National Comorbidity Survey Replication, conducted during 2001–2003, found that past 12-month substance disorders were significantly correlated with a variety of psychiatric disorders, primarily major depressive disorder, social phobia, generalized anxiety disorder, manic/hypomanic disorder, attention-deficit/hyperactivity disorder, dysthymia and intermittent explosive disorder.^2^

Comorbid disorders are more severe and chronic than single psychiatric disorders.^3^–^6^ In the general population, persons with lifetime comorbidity are more likely than those with only one disorder to experience major impairments with economic domains (e.g. unemployment, financial problems), social isolation, and interpersonal conflicts.^5^ Comorbidity is highly predictive of negative treatment outcomes.^7^,^8^ Among substance abuse patients, the severity of psychiatric symptoms is associated with poorer outcomes.^9^–^11^ Among mental health patients, particularly persons with schizophrenia, a comorbid addictive disorder has been associated with mental health treatment and medication, higher re-hospitalization and emergency room visits, homelessness, criminality and violence, suicide attempts, increased fluctuation and severity of psychiatric symptoms, legal problems, family stress, and HIV/HCV infection.^12^–^21^

## Purpose of the Study

The study’s purpose is to determine treatment outcomes for patients who present with drug use vs. those presenting with no drug use at admission to a psychiatric day treatment program with dual diagnosis capability. The analysis extends previous research on drug use comorbidity in psychiatric treatment in several ways:

- Drug use was measured by confidential research interviews and urinalysis at treatment admission and follow-up.
- Drug use and psychiatric symptoms were measured using the same procedures at admission and follow-up.
- An unselected psychiatric diagnostic sample was studied (i.e. not limited to one diagnosis such as schizophrenia or major depression).
- Outcomes were compared for psychiatric-only versus comorbid drug-using patients in psychiatric day treatment, in contrast to previous research conducted either with inpatients or with a comorbid sample only.

## Methods

### Setting

The setting was a psychiatric continuing day treatment program located in New York City. Patients in this program usually have a three times a week, half-day schedule, either in the morning or afternoon, and participate in one to four groups per day. Patients are offered breakfast and lunch on days they come to the program. The program provides mental health services for persons with single psychiatric disorders as well as for those dually diagnosed with psychiatric and substance use disorders. Specialized groups are offered for patients with co-occurring disorders, such as “Substance Abuse Awareness” “Relapse Prevention,” and a 12 Step-based dual recovery group. The program falls into the category of dual diagnosis-capable mental health treatment (but not integrated treatment).^22^

### Study sample

Two cohorts of patients newly admitted to the program were recruited as part of a larger research study, the first from March to December 2003 (n = 81) and the second from May 2004 to December 2005 (n = 148), for a total of 229 patients. Patients were referred from various mental health and drug treatment settings, including psychiatric inpatient units, mental health residences, other outpatient mental health clinics, outpatient drug abuse treatment clinics, or were self-referred through community contacts.

### Study procedures

Consecutive admissions to the program were referred by a program intake counselor to a study research assistant. Patients were excluded from study participation only if they were younger than age 18, did not understand or speak English, appeared intoxicated on drugs or alcohol, carried a diagnosis of mental retardation, were deemed actively psychotic by the clinic’s intake coordinator, or appeared unable to understand and give informed consent.

All patients who agreed to participate in the study signed an informed consent. Participants received compensation of $20 for a confidential baseline interview and biological specimens and $40 for the similar follow-up protocol six months after admission. Follow-up was attempted for all subjects even if they had left the program. The response rate at follow-up was 82%.

The study protocol was approved by the Institutional Review Boards (IRBs) of the host research site and the organization that conducted the study.

### Study measures

#### Substance use self-reports

The Drug/Alcohol Use section of the Addiction Severity Index (ASI) includes a list of drugs asking the number of days in the past 30 that each drug was used.^23^

#### Drug toxicology

Urine specimens were obtained and toxicology was conducted by on-site immunoassay (Roche TestCup) for opiates (morphine), cocaine metabolite (benzoylecgonine), marijuana (THC), and amphetamines. Urine specimens were obtained for 96.8% of the sample.

#### Colorado symptom index (CSI)

The CSI was developed specifically for assessment of symptoms at levels experienced by people diagnosed with mental illness;^24^ it has been independently validated.^25^

#### Symptom checklist-10R (SCL-10R)

The SCL-10R was developed to broadly represent both primary and secondary factors of the SCL-90; items from each of the original nine subscales of the SCL-90 are included. The SCL-10R contains six primary factor items and four additional items to include secondary factors of somatization, phobic avoidance, hostility and paranoia. The SCL-10R was developed to provide a brief measure of psychological distress that can be used with heterogeneous clinical populations; it is highly correlated with the total score of the SCL-90.^26^

#### Positive affect (PA)

This is the Positive Affect scale from the Positive and Negative Affect Schedule (PANAS); it was chosen as a measure of positive mood that differs from the negative symptom scales. The PANAS scales have satisfactory psychometrics^27^ and are widely used as a measure of mood states.

#### Quality of life (QoL)

This was measured by the Quality of Life Enjoyment and Satisfaction Questionnaire (Q-LES-Q), developed for use with patients with mental and other medical conditions as well as non-patients,^28^ that has been independently validated.^29^ The Social Relationships and Leisure Time Activities subscales were combined into a single additive index.

All the above measures were obtained at treatment admission and follow-up and were used to assess treatment outcomes. These data were obtained confidentially for research purposes and were not shared with program staff.

#### DSM-IV disorders

At admission, program psychiatrists diagnosed past 12-month DSM-IV psychiatric and substance use disorders. We report these data descriptively, but meeting or not meeting DSM-IV criteria was not employed as an outcome measure because the program does not repeat these diagnoses, 12 month prevalence would not be usable for a six-month follow-up, and changes in psychiatric status and substance use in clinical research are typically measured by comparing symptomatology between restricted time periods (e.g. 30 day windows).

#### Study definition of drug use comorbidity

Patients are defined as comorbid if they reported any use of cocaine, opiates (illicit or illicitly obtained), marijuana, or (meth)amphetamine in the 30 days prior to admission or if they tested urine-positive for one of these four classes of drugs at admission. Twelve-month DSM-IV diagnosis of drug dependence or abuse at admission was not employed to define comorbidity because such patients would not necessarily be using drugs prior to admission and moreover, there is internal evidence that many patients who are using drugs extensively (or did so within the last year) are not diagnosed because of their reluctance to disclose use to program staff at admission.^30^

#### Substance use history

A patient was classified as having a lifetime substance use history if any of the following obtained: Prior drug/alcohol treatment or detoxification episode(s); prior participation in 12 step groups for drugs/alcohol; past regular use of specific drugs or alcohol (defined as weekly or more frequent use over at least one year); meeting lifetime criteria for drug or alcohol use disorder on the M.I.N.I.^31^

### Statistical analysis

Outcomes are analyzed with contingency tables, difference of means, ordinary least squares multiple regression and multiple logistic regression. Statistical significance is set at p < 0.05 (2-tailed). The outcome analyses are based on the follow-up sample of N = 187. Changes in the use of specific drugs between admission and follow-up are presented for cocaine, opiates, marijuana and amphetamines because for these drugs the study has both self-reports and urinalysis, the latter enhancing the validity of the measures.

## Results

### Characteristics of sample

The majority (60%) of the sample was male and from minority groups, with an average age of 39 years. Most were supported by public assistance (69%), had substance use histories (93%) and had prior episodes of psychiatric treatment (90%) (Table 1).

At admission to this program, the most frequent primary psychiatric diagnosis was major depression (25%), followed by equal frequencies of bipolar (13%), other mood (13%), schizoaffective (13%) and schizophrenic (13%) disorders (Table 2).

The most frequent drugs used within 30 days before admission (based on both self-reports and urinalysis) were cocaine/crack (33%), marijuana (33%), opiates (18%), and amphetamines (6%); 61% had used at least one of these four drugs (Table 3). The amount of use among patients who reported use was substantial; the mean days used in the past 30 was 11.4 for cocaine/crack, 11.2 for marijuana and 9.6 for opiates. However, there was no self-reported use of (meth)amphetamines; all (meth)amphetamine users were classified as such by positive urinalysis. We examined how many patients were classified as drug users based solely on what could be characterized as infrequent recreational use of marijuana, which we defined as one or two days of use in the past 30; this was only 10 patients or 4.4% (10/229) of the sample.

### Drug use outcomes

For each individual drug, the percentage of patients positive at admission who remitted from drug use at six months after admission significantly exceeded the percentage negative at baseline who initiated drug use. These respective percents were 48% vs. 11% for cocaine, 35% vs. 17% for marijuana, 58% vs. 9% for opiates and 100% vs. 2% for amphetamines (Table 4). However, overall use of any of the four drugs decreased only slightly, from 59% of subjects at admission to 51% of subjects at follow-up (based on the follow-up sample of 187).

### Mental health outcomes

Overall in the sample, there were significant decreases in symptoms on the Colorado Symptoms Index and the Symptom Checklist-10R and significant improvement on Quality of Life, with no change on Positive Affect (Table 5).

Further, based on regression analyses, there were no significant differences between those identified at admission as drug users vs. non-drug users in psychological symptom reduction or in improvement on Quality of Life, controlling for the baseline value of each outcome (Table 6).

It is possible that alcohol misuse at admission might suppress a relationship between drug use and psychiatric outcomes. We constructed a variable, “alcohol intoxication days at admission,” ranging from 0–30, and entered it as a covariate in the regressions in table 6; this variable was not significant and did not alter the lack of association between substance use and clinical outcomes.

## Discussion and Conclusions

Surprisingly, there are almost no previous studies comparing psychiatric and/or drug use outcomes for psychiatric-only versus comorbid drug-using adult patients in either standard or “dual diagnosis-capable” psychiatric day treatment.^32^ Virtually all previous studies have been in primary addiction programs,^33^ or in inpatient settings,^34^–^37^ or have evaluated outcomes of integrated and other psychiatric treatments for comorbid patients only, without single disorder comparison groups.^38^

There is value in better understanding outcomes for comorbid patients in dual diagnosis-capable psychiatric programs. Simultaneous treatment for substance use disorders and serious mental illness is still infrequent; data based on the National Survey of Drug Use and Health indicates that only 15.5% of persons with both disorders receive both forms of treatment.^39^ No numeric estimate is available of what proportion of that is fully integrated treatment, but fully integrated treatment is believed to remain rare^33^,^38^ and perhaps even decreasing due to resource constraints.^33^ Thus, dual diagnosis-capable treatment is presently a more realistic service option; this study shows that patients presenting with drug use can benefit psychiatrically from such treatment to about the same degree as patients presenting without drug use.

There are only a few previous studies to which our results can plausibly be compared, although there remain many differences among the studies. A 12-month prospective follow-up was conducted of schizophrenic patients with and without a substance disorder receiving continuing mental health care, concluding that the “dual disorder patients in this cohort did no worse than the single disorder patients with respect to positive and negative psychiatric symptoms” and that the treatment “did not significantly impact levels of alcohol and drug use”.^40^ Outcomes at 8 weeks after admission to a “standard” psychiatric day treatment program were compared for patients with and without “coexisting alcohol and/or drug abuse;” the substance abusers left treatment at higher rates and had more suspensions, including for continuing abuse.^41^ Chouljian et al^42^ compared substance use outcomes over 18 months for schizophrenic outpatients with and without “problem substance use;” the overall level of substance use and problem use remained stable, while problem use of cocaine and polysubstance use increased over time. A clinical trial of citalopram for outpatients with major depression compared outcomes among several diagnostic subgroups including patients with comorbid substance use disorder only and those with no comorbid anxiety or substance use disorders; these two groups did not difference significantly on changes in depressive symptom severity. Changes in substance use were not reported.^43^ The results of these studies for psychiatric symptom change are similar to the current study, although the current study cannot examine outcomes for specific psychiatric diagnostic groups, since the sample sizes of those groups are too small. None of the programs in which the above studies were conducted appear to have been dual diagnosis-capable, which may account for the lack of influence on substance use, whereas the current study did identify a limited positive effect, at least in terms of remission.

Ours may also be the first study to report the rate of initiation of several major types of drug use among psychiatric outpatients who were abstinent from those drugs at treatment admission. Confidence in the results is increased because drug tests were included in the drug use measure, in contrast to virtually all previous outcome research in psychiatric day treatment. Underreporting of drug use in high risk populations is a pervasive problem in research.^44^

Psychiatric day treatment appears to benefit comorbid patients through substantial remission rates from cocaine, marijuana, opiate and amphetamine use by six months after admission. Yet overall drug use decreased only slightly because some patients who were abstinent at admission, initiated use of one of the index drugs by follow-up. Virtually all the patients reported substance use histories (93%) and it may be that some or all of the remaining 7% failed to disclose or forgot; thus, this sample was highly susceptible to relapse. Although relapse rates were not high for any individual drug (Table 4), because a large majority was not using any given drug at baseline, even low relapse rates led to considerable absolute numbers of relapsers.

Preventing relapse to drug use is a challenge for psychiatric day treatment, because virtually all patients may be at risk, and it is difficult to predict who actually will relapse. However, clinical attention to the risk of relapse would be facilitated if day treatment programs broadened or improved assessment procedures for determining drug use at admission. A previous paper showed that this program using typical clinical interviews identified only a fraction of the patients with drug use at admission;^30^ drug toxicologies should be considered as part of a comprehensive assessment at admission.

Psychiatric day treatment was associated with significant improvements in psychological distress and quality of life for the sample as a whole, although one measure, positive affect, did not show change. The most encouraging finding was that there were no significant differences in mental health improvements between patients who used one of four common illicit drugs at admission vs. patients who were abstinent from these drugs at admission. Although the present program was not an integrated treatment model, it was dual diagnosis-capable, e.g. providing specialized group therapy and peer support groups that address comorbidity issues, which may help account for these encouraging results.

## Figures and Tables

**Table 1:** Sample characteristics at admission to treatment (n = 229).

Male	60%
Hispanic	41%
Black	42%
White	18%
Currently employed	3%
Public assistance	69%
Unstable housing	16%
Substance use history	93%
Ever received psychiatric treatment	90%
Age in years (mean, sd)	39 (9.1)

**Table 2:** DSM-IV Axis I diagnoses – 12 month (n = 229).

Major depression	25%
Bipolar	13%
Other mood disorders	13%
Schizoaffective	13%
Schizophrenia	13%
Psychotic disorders NOS	7%
Anxiety disorders	3%
Other disorders	13%
Cocaine	11%
Opioids	11%
Marijuana	8%
Sedatives	4%
Polysubstance	11%
Any drug	34%
Alcohol	11%

**Note:** Substance use diagnoses represent one of up to three disorders recorded by program psychiatrists; other diagnoses represent the primary diagnosis.

**Table 3:** Drug use at admission—positive self-report or urinalysis (n = 229).

Cocaine	35%
Marijuana	33%
Opiates	18%
(Meth)amphetamines	6%
Any of these 4 drugs	61%

**Table 4:** Changes in drug use (N = 187).

	Positive at admission who remitted at follow-up	Negative at admission who initiated by follow-up	P-value
Cocaine	48% (30/62)	11% (14/125)	<0.001
Marijuana	35% (20/58)	17% (22/129)	<0.01
Opiates	58% (21/36)	9% (14/151)	<0.000
(Meth) amphetamines	100% (11/11)	2% (4/176)	<0.000

**Table 5:** Changes in mental health (N = 187).

	Admission M (SD)	Follow-up M (SD)	P-value
Colorado symptoms index	2.64 (1.10)	2.31 (0.97)	<0.000
Symptom Checklist-10	1.75 (1.05)	1.46 (0.99)	<0.000
Positive affect	2.99 (1.10)	2.99 (0.98)	NS
Quality of life	3.32 (0.69)	3.43 (0.69)	<0.05

**Table 6:** Effect of drug use at admission on mental health outcomes.

Outcome (dependent): Colorado symptoms index (CSI)				

Predictors	B	SE B	T	P-value
Drug use at admission	0.044	0.119	0.37	0.71 (NS)
CSI at admission	0.556	0.058	9.57	<0.000
**Outcome (dependent): Symptom checklist -10 (SCL)**				
Drug use at admission	−0.041	0.116	−0.35	0.73 (NS)
SCL at admission	0.589	0.055	10.81	<0.000
**Outcome (dependent): Quality of Life (QoL)**				
Drug use at admission	−0.081	0.094	−0.86	0.39 (NS)
QoL at admission	0.417	0.067	6.25	<0.000
